# Beyond the Gut: Unveiling Methane's Role in Broader Physiological Systems

**DOI:** 10.1096/fba.2025-00036

**Published:** 2025-08-26

**Authors:** Matthew Kerr, Madeleine Ball, Nabeetha Nagalingam, Rui Pinto‐Lopes, Max Allsworth, Billy Boyle

**Affiliations:** ^1^ Owlstone Medical Ltd. Cambridge UK

**Keywords:** biological marker, breath test, methane, physiology, translational research

## Abstract

Interest in the endogenous role of methane has grown rapidly over the past decade, driven both by its relevance for disease detection (including intestinal methanogen overgrowth) as well as discoveries that raise the possibility of endogenous sources of methane and suggestive evidence of methane effects relevant to physiology. This review explores both established and emerging origins of breath methane, its physiological relevance, and the evolving landscape of detection methods. We aim to summarize current understanding and provide a platform to outline key directions for future research. Evidence supports the existence of non‐microbial, endogenous methane production pathways and potential biological effects beyond the gut. However, the concentrations generated via these pathways and the levels required to elicit physiological responses remain under investigation. Recent technological advances have enabled more accessible and longitudinal breath methane monitoring, opening new avenues for research and clinical application.

## Graphical abstract

This figure summarizes key themes of the review, highlighting methane's dual origins from gut methanogens and endogenous oxidative processes, the principal analytical methods for breath detection (GC‐FID, infrared spectroscopy, and metal oxide sensors), and its proposed physiological effects. These include modulation of gastrointestinal motility, inflammation, short‐chain fatty acid (SCFA) production, and energy regulation. Together, these aspects underscore the potential systemic significance of methane beyond the gut.

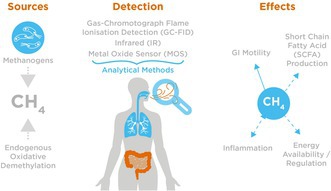

## 1 Introduction

Methane has long been regarded primarily as a greenhouse gas; however, we are now beginning to appreciate that it may also play important roles in human physiology. Traditional views hold that in the setting of human biology, methane is primarily produced by methanogenic archaea in the gastrointestinal tract of individuals harboring these microbes. This methanogen activity is understood to influence local digestive processes and has more recently been reported to potentially impact broader physiological functions, including immune modulation and oxidative stress responses.

In addition to novel functions, recent studies have identified further potential sources of methane production, challenging these traditional views that its origins are solely microbial. Understanding the diverse origins and physiological effects of methane is critical to better understand its implications for health and disease.

In this review, we explore the origins, effects, and clinical implications of methane in human physiology. By synthesizing current research, we aim to better understand the mechanisms underlying methane production as well as the potential impact of these processes on gastrointestinal function, immune modulation, and metabolic pathways.

## 2 Sources of Methane in Human Breath

### 2.1 Gut Microbiome

The gastrointestinal (GI) microbiota encompass a diverse community of microorganisms inhabiting the human gut. The fermentation of dietary fibers and metabolism of endogenous compounds by these gut microbes produce a wide range of volatile products. Some of these volatiles have already seen widespread interest, including the short‐chain fatty acids (SCFAs) acetate (C_2_), propionate (C_3_) and butyrate (C_4_). These SCFAs are normally produced at a ratio of around 60:20:20, with 500–600 mmol produced per day [1, 2, 3]. However, these SCFAs form a relatively minor part of the ~0.2–1.5 L of gas produced per day by the gut microbiota of most healthy people [4, 5, 6]. The gases responsible for the majority of this volume are hydrogen (H_2_), carbon dioxide (CO_2_), and methane (CH_4_), together contributing more than 99% of the intestinal gas volume [7] as well as various sulfur‐containing trace gases, including hydrogen sulfide (H_2_S), methanethiol (CH_3_SH), and dimethyl sulfide ((CH_3_)_2_S), which arise from protein fermentation [8] and contribute to the final 1%.

The gut microbiome constitutes the oldest and most extensively studied source of methane production in humans. At a composition level, methanogenic archaea are recognized as the primary producers of methane through anaerobic metabolism. These methanogens utilize the methylotrophic pathway, reducing CO_2_ with H_2_ or formate to form CH_4_. This process occurs primarily in the colon and to a lesser extent in the small intestine. There is comparatively little diversity regarding specific methanogen species, as illustrated in Table 1.

**TABLE 1:** Illustrating the four key archaeal species associated with methane production within the GI tract.

Phylum	Genus/species	Gas	References
Euryarchaeota	*Methanobrevibacter smithii*	CH_4_	Weaver et al. [9]
	*Methanosphaera stadtmanae*	CH_4_	Fricke et al. [10]
	*Methanobrevibacter oralis*	CH_4_	Scanlan et al. [11]
	*Methanomassiliicoccus luminyensis*	CH_4_	Nkamga et al. [12]

The predominant species (across both healthy and diseased states) is *Methanobrevibacter smithii* , with *Methanobrevibacter stadtmanae* occurring to a lesser extent [9, 10, 12]. Supporting their integral role in methane production, the levels of these archaea can be seen to reflect levels of overall methane production, with the microbiomes of people classed as high methane emitters (CH_4_ > 5 ppm) characterized by a 1000‐fold increase in *M. smithii* [13] compared to low methane emitters.

The abundance of methanogens demonstrates not only inter‐individual variation, but also correlations with external factors, such as age and diet. Multiple studies have reported age‐related shifts in the gut microbial ecosystem, which favor methane production with increased age [14, 15, 16]. Although less extensively studied, dietary influences on methanogen abundance have also been observed, with Methanobrevibacter levels negatively correlated with the intake of total fat, saturated fat, and omega‐3 fatty acids [13]. Whilst the absolute abundance of methanogens is a key determinant of the rate of methane production, substrate availability, via exogenous dietary consumption, is another important factor. For example, vitamin B12 deficiency has been linked with altered methane production via the modulation of formate availability [13].

Once generated in the gut, methane is able to diffuse across the intestinal mucosa into the portal circulation, where it undergoes gas transfer in the alveolar space and is subsequently exhaled [17]. It is estimated that 20%–50% [18, 19] of the methane produced in the gut is excreted via exhaled breath. This enables breath methane measurement to serve as a noninvasive approach for the clinical assessment of gastrointestinal health and the investigation of methane‐related disorders.

### 2.2 Methane's Role in Medical Diagnostics

Whilst the focus of this review is on exploring the extra‐gut production, roles, and measurement of methane, it is important to also consider its established clinical application in the diagnosis of intestinal methanogen overgrowth (IMO) [20]. Unlike traditional small‐intestinal bacterial overgrowth (SIBO), IMO represents the overgrowth of methanogenic archaea rather than bacteria in either the colon or small intestine [20]. Diagnosis for both SIBO and IMO is based on a breath test, assessing exhaled levels of H_2_ and CH_4_.

Diagnostic thresholds have evolved over time, but methane levels on‐breath greater than 10 ppm are now widely accepted as indicative of IMO [21] with some classifications further considering baseline breath methane levels greater than 1–3 ppm as elevated [22]. The reported prevalence of IMO varies across studies, but recent data suggest that around 60% of patients with SIBO also present with elevated methane levels (47.3% with both elevated hydrogen and methane and 12.4% with only elevated methane levels) [23].

SIBO and IMO are inherently related, but the delineation between the two is important given there are both different treatment recommendations and reported physiological differences between them. SIBO responds well to rifaximin, whereas archaea associated with IMO are resistant to most antibiotics and respond better to combination therapy (e.g., rifaximin/neomycin) compared with a single antibiotic (e.g., rifaximin alone) [20, 24]. At a physiological level, IMO has been seen to correlate with delayed small bowel transit and colonic transit compared to SIBO [25] which will be discussed further later in this review.

The two conventional hydrogen‐methane breath tests for SIBO use accurate and precise, offline, laboratory‐based techniques. While this approach is effective in a diagnostic setting, it is not easily adaptable for longitudinal testing. The single‐timepoint nature of these assessments makes it challenging to capture real‐time changes in hydrogen and methane levels, limiting the ability of individuals to track or respond to fluctuations over time [26]. Recent developments in the field of breath methane monitoring, including clinically accepted breath methane monitoring devices [27], allow for longitudinal measurements of breath methane and the establishment of a personalized baseline to guide further testing. These measurement systems for methane will be further discussed in this review.

### 2.3 Human Endogenous Processes

**FIGURE 1:**
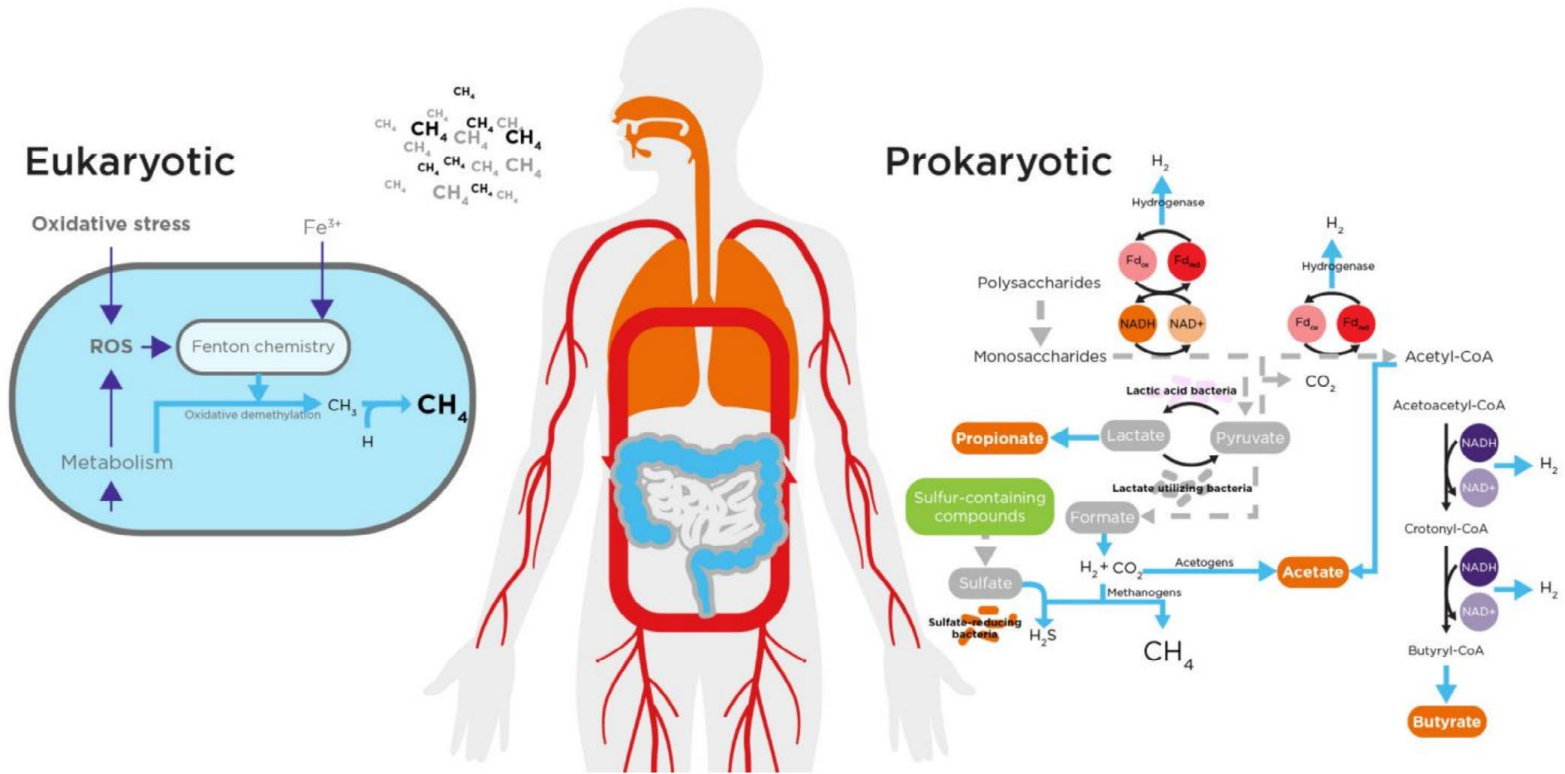
Demonstrating the proposed routes for both exogenous and endogenous methane production. Endogenous methane production, by contrast, relies on oxidative demethylation of methane‐containing moieties by reactive oxygen species. Exogenous methane production relies on the generation of methane by prokaryotic species in anaerobic conditions within the GI tract.

In addition to the relatively well‐characterized production of methane by methanogenic archaea in the gut, emerging data have demonstrated, across in vitro and in vivo settings, that there may be additional host‐derived endogenous sources that could contribute to measurable methane levels, particularly in settings of stress. These studies suggest that elevated levels of reactive oxygen species (ROS) can produce methane, as illustrated in Figure 1. This generation would rely on the Fenton reaction to produce hydroxyl radicals from hydrogen peroxide (H_2_O_2_), and subsequent oxidative demethylation of methylated sulfur or nitrogen compounds (e.g., methionine, dimethyl sulfoxide, or trimethylamine).

Methane production as a result of oxidative stress in vitro has been observed, with methane being formed after the application of 2 M H_2_O_2_ to a variety of endogenous compounds. Of these compounds, choline chloride was the most potent, generating 4–25 μM methane, but methionine and ethanolamine were also capable of producing measurable amounts of methane [28, 29]. Of note, in these settings, compounds that generated appreciable concentrations of methane also demonstrated some antioxidant activity, with reductions in the generation of ROS [29].

Beyond in vitro systems, methane production has also been demonstrated ex vivo through the application of oxidative stress (via H_2_O_2_ and ascorbic acid) to isolated mitochondria. In this setting, oxidative stress induced methane production, the rate of which increased proportionally with both the level of reactive oxygen species (ROS) and the amount of mitochondrial protein added. Levels of production at 100 mM H_2_O_2_ and pH 7.4 reached 0.3 nmole methane per mg mitochondrial protein per 60 min [29] and the application of catalase prevented these effects, lending further weight to oxidative stress driving this methane production [28]. To assess the potential physiological relevance of these findings, it is helpful to compare these production rates with those observed in humans. Using the liver as an example organ, and extrapolating from these experimental rates of production, one can obtain approximate estimated rates of methane production between 58 and 118 μmole from a liver in 60 min (taking the average liver weight of between 968 and 1860 g [30], of which around 20% is mitochondria by volume [31]). Making the approximation of negligible loss during this time, and a blood volume of 5 L, one could estimate blood concentrations between 11.6 and 23.6 μM placing values within an order of magnitude of predicted levels in blood of around 2 μM in blood under normal conditions [32].

Moving from isolated mitochondria to a cultured endothelial cell setting, Adamczuk et al. [33] demonstrated that cultured cells produce methane even at baseline (2 nmol/mg), and that this could be increased by exposure to agents associated with elevations in ROS such as sodium azide (NaN_3_) (15 nmol/mg) or 2,4‐dinitrophenol (DNP) (23 nmol/mg) [29]. This phenomenon has been observed both in mammalian cells as well as plant cells, with grapevine demonstrating a similar increase in methane production following exposure to NaN_3_ [34].

The first evidence supporting the translation of these in vitro and ex vivo findings to an in vivo setting was provided by Tuboly et al. [35], who demonstrated elevated methane production in rodents following NaN_3_ administration. This was further supported by evidence from Keppler et al. [36], first identifying the production of methane from leaves (0.3 ng/g dry weight) and then from humans, demonstrating methane release from radiolabeled methionine both in blood and from the skin (headspace) [37]. Two additional aspects of these experiments stand out as providing potential further insights into this phenomenon. First, Tuboly et al. demonstrated that this elevation in methane could be prevented by co‐administration of α‐glyceryl phosphorylcholine, a protectant against lipid peroxidation, further supporting that methane production is associated with oxidative stress. Second, both Tuboly et al. and Keppler et al. took steps to remove microbial‐linked methane production, either via the administration of rifaximin or UV irradiation (respectively) providing a good evidence base that observed effects were driven by extra‐microbial production of methane.

The relevance of these findings to human physiology had its first indications in 2013, when Tuboly et al. [38] demonstrated that administration of lipopolysaccharide (LPS) in mice (an acute sepsis setting) corresponded with a 2–3 fold increase in methane production. These data suggested that infection, and associated elevations in inflammation/oxidative stress, may provide a real‐world setting for elevations in non‐microbial methane production. While further work validating this finding in sufficiently powered studies is required, there are preliminary indications that this may hold for human infection as well, with Keppler et al. [39] demonstrating elevations above baseline (on a similar order of magnitude to Tuboly) in response to COVID‐19.

## 3 Detection and Measurement of Methane in Breath

### 3.1 Breath Sampling and Analytical Techniques

Having reviewed routes for methane formation in humans, we will now focus on measurement mechanisms. As previously discussed, breath methane measurement is already commonly employed in the diagnosis of SIBO and IMO. The two most widely used analytical techniques in this setting are GC‐based systems and IR spectroscopy. Both are well‐established, reliable laboratory‐based analytical tools with clearly defined performance characteristics.

Gas chromatography requires compressed gases to pass the sample through a separation column and then to a flame ionization detector and thermal conductivity detector for measurement of methane and hydrogen, respectively. As standard, this equipment is bench mounted and requires trained operators. Commercially available on‐breath methane monitoring devices based on infrared spectroscopy are typically more portable benchtop devices, such as the Gastrogenius Breath Monitor (Laborie). The sensitivity of infrared spectroscopy can be increased by multiple passes of the infrared light within an optically reflective chamber. This arrangement requires a comparatively expensive optically resonant chamber with a light source, reflective surface, and detector mounted within.

A suitable technology that presents low cost, low power, and sufficient sensitivity can be realized in metal oxide sensors (MOS). These electronic devices are typically mounted in standard electronics component packages (e.g., TO‐5 cans or LGA‐8 packages) and are amenable to widely available electronic manufacturing processes. However, they are inherently non‐specific, responding to a broad range of oxidizing or reducing gases. Against a complex matrix such as human breath, this can pose challenges related to sensitivity. Additionally, MOS are sensitive to both changes in humidity and temperature, and both of these environmental parameters can change dramatically during a single exhalation.

These on‐breath VOCs and environmental considerations present as interferents and can introduce an unacceptable error into the estimates of methane and hydrogen concentrations. There are several approaches to overcome these limitations, the first being mathematical compensation for the environmental interferents, which can be readily measured and compensated for, such as temperature and humidity. The second is through the use of molecular filters and adsorbents that preferentially retain or retard the diffusion of larger, lower vapor pressure compounds. This latter point is similar in concept to the stationary phase of a GC column retaining certain compounds over others. This approach enables the use of simple, low cost, low power MOS devices to operate as both sensitive and selective devices that are well matched to the technical requirements for continuous real time monitoring of exhaled methane and hydrogen. Employment of these approaches has been demonstrated to result in comparable data quality between benchtop IR (Gastrogenius Breath Monitor) testing and handheld MOS sensor (OMED device) testing [27].

**FIGURE 2:**
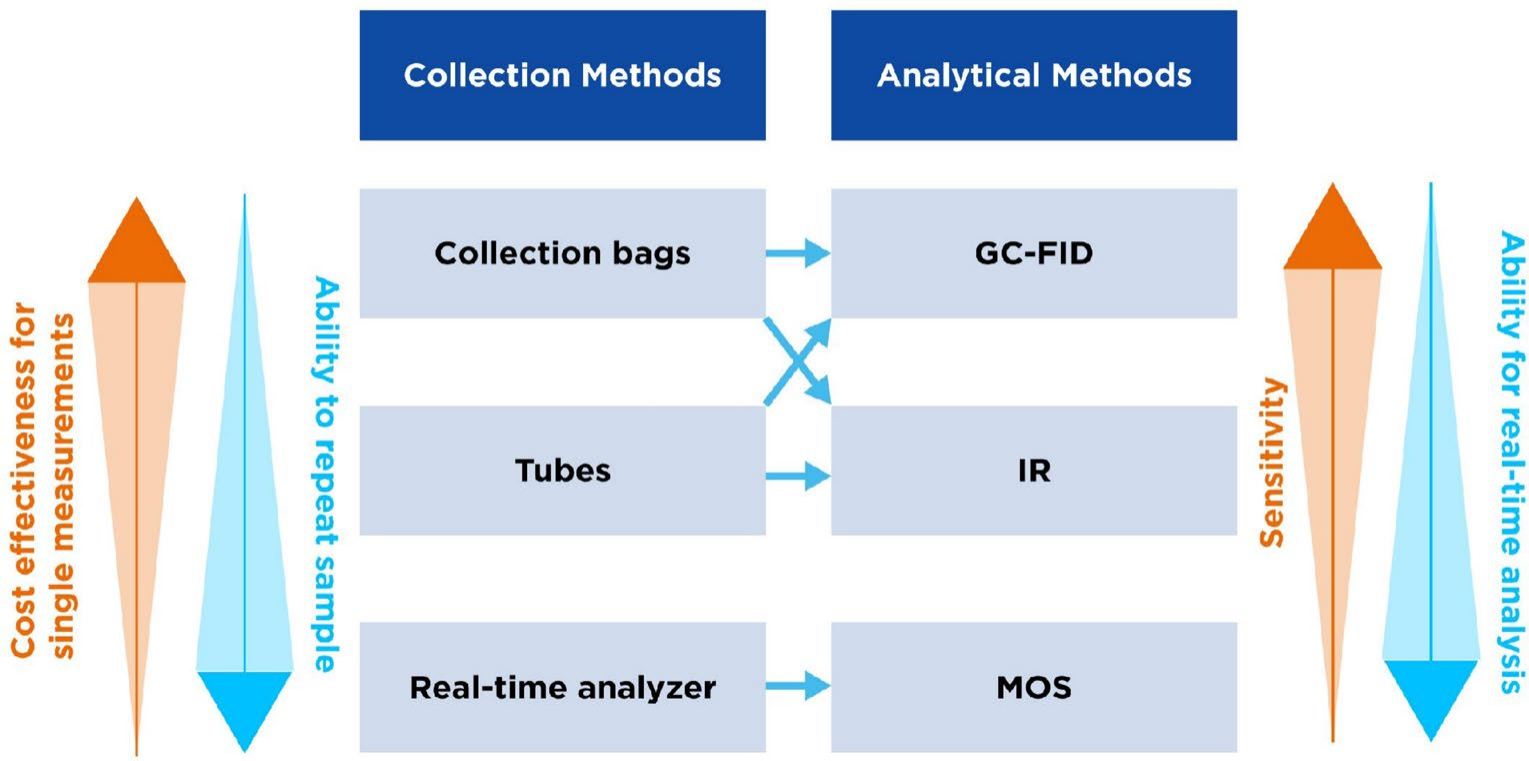
Summarizing the advantages and limitations of the collection and analytical methods used.

The relative advantages and limitations of these collection methods (Table 2) and analytical methods (Table 3) are summarized below in Figure 2.

**TABLE 2:** An overview of collection methods for breath methane analysis.

	Collection bags	Tubes	Handheld real‐time analyzer
Overview	One of the most common methods used, where patients exhale directly into a bag	Uses tubes through which the patient exhales.	Involves the use of portable devices that analyze breath in real‐time with no need for separate sample collection/storage
Different forms	Mylar bags (made from a type of polyester film impermeable to gases) or Tedlar bags (made from PVF)	Vacuum tubes (draw in the breath sample automatically) or glass/plastic tubes	The OMED Health Breath Analyzer [27] and the foodmarble AIRE 2 [40]
Procedure	Patients take a deep breath and exhale completely into the bag. The bag is subsequently sealed for later analysis	The patient exhales through a mouthpiece connected to the tube, which is then sealed after collection	Patient breathes directly into the analyzer through a mouthpiece. Gas concentrations are fed back in real‐time
Advantages	Simple and cost‐effective	Easy to use and transport	Provides instant results and allows for simple repeat measures
Considerations	Bags can be challenging to handle post‐collection and can lead to sample contamination/loss Possible inaccuracy due to surface adsorption of low vapor pressure compounds	Tubes may require specific storage to prevent sample degradation Requires measurement of CO_2_ for compensation of dilution effects or sample loss	Device calibration is crucial for accurate readings. Requires methods for increasing selectivity and specificity

**TABLE 3:** An overview of analytical methods for breath methane analysis.

	GC‐FID	IR	MOS
Overview	GC‐FID combines GC for separation of components of a breath sample and flame ionization detection for methane and quantification	IR measures the absorption of IR light by methane to determine its concentration	MOS detects gases based on changes in electrical resistances of a metal oxide sensor when it interacts with methane
Advantages	High sensitivity, high specificity, and quantitative analysis	Real‐time analysis and easier than GC‐FID	Cost‐effective, durable and provides real‐time analyses
Limitations	Complexity and cost, requiring sophisticated equipment and trained personnel. Time‐consuming, requiring laboratory preparation	Unlikely to reach the level of GC‐FID for low concentrations. Subject to interference from other gases/water vapor if not properly calibrated Requires precision engineering of optical cavity, reflector, and detector	Unlikely to reach the level of GC‐FID for low concentrations. Requires appropriate calibration Broadly selective and responds to a wide range of reducing and oxidizing gases Sensitive to changes in ambient humidity and temperature Polysiloxanes can cause irreversible changes in sensitivity

## 4 Potential Effects of Methane

**FIGURE 3:**
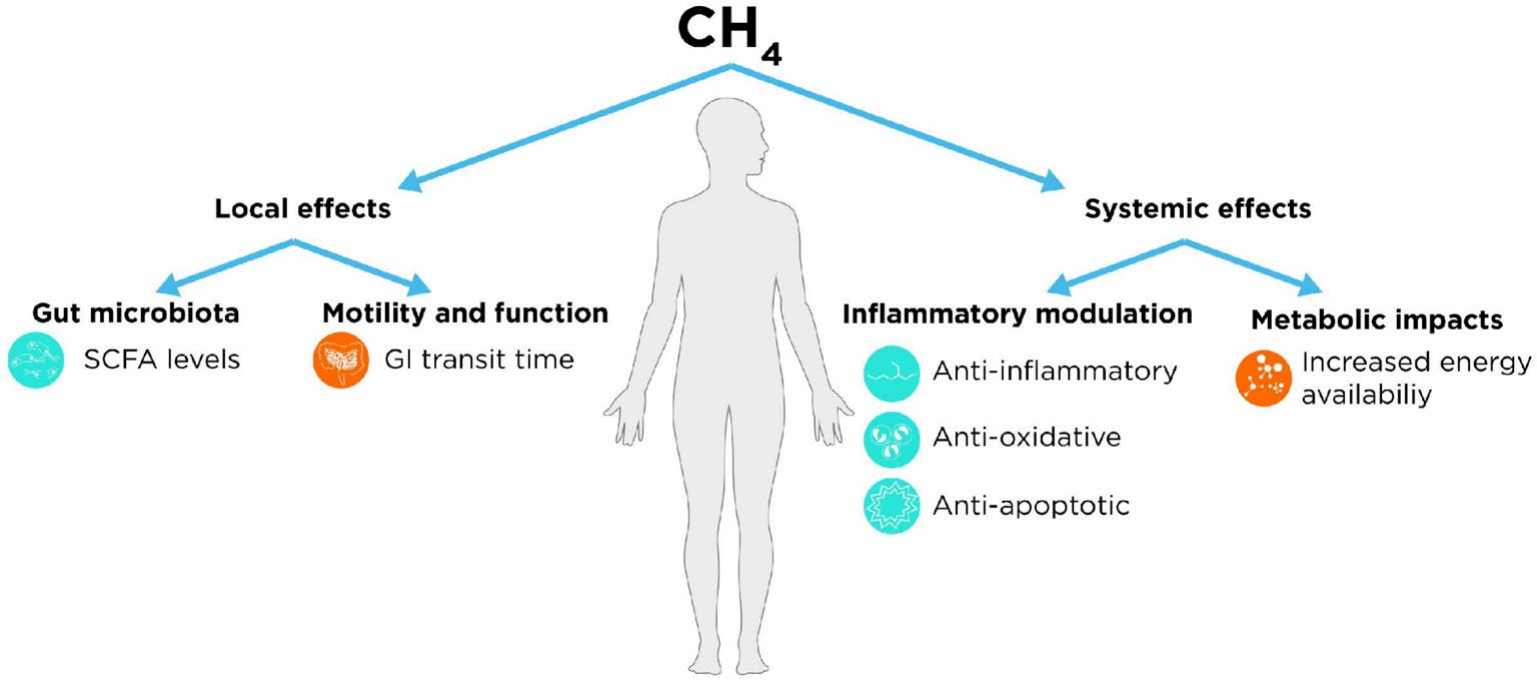
Summarizing both the local and systemic effects proposed for methane within the field. Green icons indicate an effect with positive associations; orange icons indicate an effect with negative associations.

With both the potential routes for methane production considered, as well as the value and mechanisms for testing methane concentrations on breath, the next section will focus on the local and systemic potential effects of methane, with an emphasis on delineating correlation from causation. The conclusions are summarized in Figure 3.

### 4.1 Local (GI) Effects

#### 4.1.1 Motility and Function

Data from animal studies suggests that methane can have a profound impact on GI transit time. In experimental settings, exogenous methane gas applied ex vivo has been shown to directly inhibit intestinal transit by 59% in dogs [41] and decrease peristaltic velocity in guinea pigs [42]. This can be translated to observations in human populations, with methane levels correlating strongly with slower intestinal transit times [43, 44, 45, 46, 47]. Work from Soares et al. supplemented these findings with additional detail, demonstrating that total colonic transit time averages 80.5 h in methane producers compared to 61.0 h in non‐methane producers and providing a breakdown of these transit times. This revealed substantial delays in specific sections of the colon: 17.5 h versus 10.5 h in the right colon, 29.5 h versus 10.5 h in the left colon, and 31.5 h versus 27.0 h in the rectosigmoid region [48].

These data universally support a link between elevated methane production and increased GI transit times; however, data so far have been limited to correlations. Work from Pimentel et al. extended these findings towards an in vivo model through direct administration of methane (via intestinal fistulae). This model removed other potential confounders (such as dietary effects) which may have impacted previous human studies, demonstrating a 59% increase in transit times in the presence of methane (at a concentration equivalent to a breath methane level of 50 ppm) [41]. The work of Park et al. provided the first potential mechanism to explain these observations. Namely, they identified in studies involving the infusion of methane under electrical field stimulation that methane increased the amplitude of ileal contractions across all tested frequencies (1–16 Hz) [49].

#### 4.1.2 Interaction With Gut Microbiota

The gut microbiome is inherently synergistic, and so it is reasonable to hypothesize that in methane producers, with high methanogenic archaeal levels, there may be other changes to gut microbiota composition or products. Indeed, data from Kumpitsch et al. [13] identified that high‐methane producers (> 5 ppm) demonstrate a significantly higher alpha diversity and substantially different microbiome composition compared to low‐methane producers. Methane‐emitting microbiomes were significantly associated with Euryarchaeota (Methanobrevibacter) as well as signatures of Christensenellaceae R7 group, Ruminococcus/Ruminococcaceae, Holdemanella, and the Eubacterium ruminatium groups [13], groups which are associated with dietary fiber degradation. These data support findings that when Christensenella and Methanobrevibacter are co‐grown in vitro, they form dense flocs whereby the H_2_ generated by the Christensenella supports CH_4_ production by Methanobrevibacter. In this setting, SCFA production is shifted more towards acetate and away from butyrate [50]. Supporting this, high methane producers also show increased levels of formate and acetate in the gut, with these metabolites strongly correlated with dietary habits such as vitamin, fat, and fiber intake.

This association has been investigated in vivo by a number of groups, with seemingly conflicting results. Early work in 1984 found no significant difference between the levels of SCFAs in the feces of methane producers compared to non‐methane producers [51], which was corroborated by serum findings in 1998 [52]. Subsequent work, however, found significant elevations in both fecal and serum SCFA levels (particularly in propionate, formate, and acetate) in methane producers compared to non‐methane producers [13, 53, 54] with the latest data from Fernandes et al. [55] identifying a negative correlation between breath methane levels and fecal SCFA levels in patients.

These results at first glance appear conflicting; however, when the impact of confounders is considered, namely those that may independently correlate SCFA and breath methane levels (e.g., sex, age, or diet) a trend emerges. When these results are considered within the context of age, which is known to correlate with both increased methane production [56] and decreased SCFA levels [57], we can observe that studies with an age mismatch [52, 55] demonstrate no change or a decrease in SCFA levels with elevated methane, whilst those that correct for age [13, 54] clearly demonstrate elevations in SCFA levels with elevated methane levels. It is noted that, whilst considerations of microbiome level implications of methanogen presence must be considered, these findings are supported from a purely biochemical standpoint whereby removal of H_2_ by methanogens would be expected to modify, and potentially increase SCFA production through end‐product removal [58, 59].

### 4.2 Systemic Effects

Much of the work around methane's potential bioactivity, and the focus of this review so far, has been around the potential local effects of methane in the GI system. However, data have emerged, largely via the exogenous application of methane, supporting additional potential systemic effects. These include potential activity as an anti‐inflammatory, anti‐apoptotic, antioxidant, or metabolic regulatory molecule. In this section, we will focus on some of these effects and their context.

#### 4.2.1 Inflammatory Modulation

The most common systemic effect attributed to methane is its potential as a cytoprotective compound. Studies have associated methane with three potential cytoprotective effects.

1. Anti‐inflammatory effects that manifest as reductions in TNFα, IL‐6, and IL‐1B levels following intraperitoneal (IP) dosing of methane‐rich saline (MRS). These effects appear to be mediated via IL‐10 and upstream through the PI3K‐AKT‐GSK‐3B pathway [60, 61, 62, 63, 64, 65, 66, 67, 68, 69, 76].
2. Anti‐oxidative effects, presenting as reductions in MDA or 8‐OHdG levels, as well as the prevention of loss of antioxidant activity (SOD/CAT levels) [62, 63, 64, 65, 66, 67, 68, 70, 71, 72, 73, 76].
3. Anti‐apoptotic effects, manifesting as reductions in TUNEL staining, as well as reduced caspase 3/9 activation [63, 64, 65, 66, 70, 71, 74, 76].

These effects have been observed across a wide range of diseases, including ischemia/reperfusion injury [70, 71, 72], inflammatory disease [60, 61, 62, 75] and neuronal disease [68, 76].

It should however be noted that these studies generally leverage methane‐rich saline (MRS) (at 0.99 mM), first used by Ye et al. [63], with doses between 0.5 and 20 mL/kg demonstrating efficacy (with rough end‐dosage of around 9 μmol/kg). Assuming a total blood volume of a rat at ~64 mL/kg, full displacement of methane into the blood, and minimal methane loss, this would be expected to give ~140 μM, or around 70× the levels expected from microbiome production and 14× levels expected from endogenous production during sepsis. Therefore, the dose‐dependent observation of effects in these studies brings into question comparisons between observations within these MRS dosing experiments and their impact in a real‐world setting.

#### 4.2.2 Metabolic Impacts

There has been a focus on gut dysbiosis within obesity for over 20 years now, and early work from Turnbaugh et al. [77] demonstrated that the gut microbiomes of obese (*ob/ob*) mice have increased representation of archaea compared to their control weight (*ob/+*) littermates. This was attributed to an increased ability to degrade polysaccharides, a phenomenon which was demonstrated to be transmissible, resulting in greater weight gain in lean germ‐free mice following fecal microbiome transplant [77]. Supporting increased energy harvesting driving this phenomenon, data demonstrated that co‐colonization of mice with the symbiotic pairing of *M. smithii* and *B. thetaiotaomicron* resulted in significantly greater adiposity compared with colonization of either organism alone [59].

Given the known and well‐demonstrated association of dysbiosis with metabolic syndromes [78], data surrounding correlations between methane and BMI must be approached cautiously. Despite this, there are two effects of methane that could be expected to contribute towards additional weight gain and therefore provide a rationale for a positive correlation between BMI and methane production. Namely, slowed GI transit time, providing greater time for nutrient absorption across the GI tract, and increased production of SCFAs increasing calorie availability from food (responsible for ~10% of calorie availability in humans [79]).

Translating this to a real‐world setting, the majority of data support a correlation between elevated methanogen presence and therefore breath methane levels and a higher BMI. This has been demonstrated at baseline in obese patients, where those with breath CH_4_ > 3 ppm display a BMI ~7 higher than those without [80] as well as in obese compared to lean children [81]; and, although not reaching statistical significance (potentially due to study power) also by Fernandes et al. [52]. Of note, in addition to baseline levels, correlations have also been observed between elevated methane levels following a lactulose challenge and BMI, firstly by An et al. [82] and also by Mathur et al. [83] who demonstrated a correlation only if both breath methane and hydrogen were elevated.

Despite this evidence for a positive correlation between methane and BMI, there is some disagreement amongst the field. Ozato et al. [84] found no significant difference between methane and BMI but demonstrated a lower visceral fat area in methane producers vs. non‐producers. Wilder‐Smith et al. [85] even found that people who had detectable methane in their breath following a lactose/fructose challenge had a lower BMI compared to non‐methane producers. Of note, a key difference here was that Wilder‐Smith et al. were the only group to study specifically patients with a functional gut disorder (irritable bowel syndrome, as diagnosed by Rome III criteria). On balance, the data above suggest that in the general population, higher breath methane levels are associated with a higher BMI; however, in a subset of people with functional gut disorders, this may not hold to be true, potentially due to the presence of additional factors that drive methane levels.

Following from this, methane producers also had worse glucose tolerance compared to non‐methane producers [86], and pharmacologically reducing breath methane (through antibiotic use) in obese patients improved glucose tolerance [87]. Patients who were positive for both methane and hydrogen also displayed reduced (prorated) percentage changes in BMI following bariatric surgery [88]. Whilst these data suggest that methane correlates with increased BMI and altered glucose handling, there is also data suggesting an overall potentially cardioprotective effect of methane. Wu et al. [89] found that the transition from pre‐diabetes to type 2 diabetes was associated with a downregulation of bacterial methanogenesis. Ozato et al. [84] also found that higher methane levels were associated with decreased visceral fat area, a key contributor for cardiometabolic risk. Finally, Laverdure et al. [90] found that, in an in vitro setting, GLP‐1 secretion could be stimulated by methane.

### 4.3 Potential Role of the Vagus Nerve and Cholinergic Pathway

To date, this review has explored the potential sources and physiological effects of endogenous methane. However, the mechanisms underlying some of these observed effects remain largely unknown. In this section, we highlight an emerging area of interest: the potential interaction between methane and the vagus nerve.

The vagus nerve, the longest and most extensively distributed autonomic nerve, originates in the brainstem and extends through the neck into the thoracic and abdominal cavities. This nerve carries both motor and sensory fibers, providing innervation to numerous systems and influencing critical aspects of human physiology, including heart rate, blood pressure, sweating, digestion, and even vocalization [91].

**FIGURE 4:**
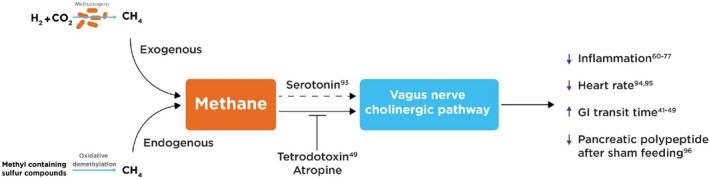
Highlighting the potential role of the vagus nerve/cholinergic pathway in mediating the effects of methane, with focus on potential regulators of this pathway, as well as shared effects.

Evidence supporting the role of methane in modulating vagal nerve/cholinergic pathway activity was first demonstrated by Park et al. [49] who identified that the application of tetrodotoxin or atropine can abolish methane‐induced increases in contraction amplitude in guinea pig ileal muscle strips. It can be noted that whilst not directly investigated further, there is data supporting that this interaction may occur indirectly, via associated changes in serotonin production [92]. Supporting the implication of the vagus nerve, the effects of methane appear to correlate closely with the outcomes associated with vagal nerve and cholinergic pathway activation. This relationship is evident in the shared anti‐inflammatory effects [60, 61, 62, 63, 64, 65, 66, 67, 68, 69, 76], alterations in heart rate [93, 94], modifications in gastrointestinal transit time [41, 42, 43, 44, 45, 46, 47, 48, 49], and the secretion of pancreatic polypeptide following sham feeding [95], as summarized in Figure 4 below. Whilst this preliminary data supports a potential role of the vagus nerve in mediating effects of endogenous methane, significant further work is required in this area.

## 5 Clinical Implications and Future Research

Breath methane measurements have received attention in the past as part of their use in the clinical diagnosis of various gastrointestinal conditions, such as SIBO. These tests largely involve fasting (to minimize baseline sample variance) followed by administration of a challenge substrate (e.g., lactulose, glucose, or fructose) and subsequent breath measurements at timed intervals.

These challenge tests help to pinpoint changes in methane levels that specifically originate in the gastrointestinal tract, allowing breath methane to be used as a test for SIBO without interference from other potential sources of methane. In contrast, using endogenously generated methane as a biomarker presents new challenges. Longitudinal breath sampling, aimed at establishing individualized baselines and detecting deviations over time, may help mitigate variability and account for confounding influence, thereby improving interpretability in non‐gastrointestinal contexts.

This approach mirrors a growing shift from static to dynamic monitoring paradigms across medicine. This has been reflected in assays such as blood glucose monitoring, where technological advancements have brought with them a shift from static fingerstick blood glucose readings to the widespread use and adoption of continuous glucose monitoring. Technological advances in breath analysis now enable real‐time data collection outside of clinical environments, opening the door for more granular, longitudinal studies. This may facilitate exploration of methane's relationship to lifestyle factors (e.g., diet, physical activity, and BMI), microbiome shifts, infection, or therapeutic interventions.

The field is thus well‐positioned to explore breath methane beyond gastrointestinal disorders, including its potential associations with systemic health and disease states. These developments create opportunities to investigate links with inflammation, redox balances, and physiological responses in real‐world populations.

## 6 Conclusion

In biological systems, methane has traditionally been viewed as a byproduct of microbial activity within the gastrointestinal tract. Emerging evidence challenges this view in two key ways: first, by suggesting potential additional routes of endogenous production in the form of oxidative demethylation, and second, by proposing novel physiological effects, ranging from local gastrointestinal changes to possible roles in the regulation of systemic inflammatory processes.

These potential alternative sources and wider effects of methane raise interest in its development as a potentially clinically relevant biomarker or therapeutic outside of immediate gastrointestinal settings. However, determining whether these associations are causal, elucidating their underlying mechanisms, and establishing whether they occur at physiologically relevant concentrations remain important priorities for future research.

Accurate and accessible measurement remains a key consideration in the study of breath methane. Techniques such as gas‐chromatography flame ionization detection, infrared spectroscopy, and metal oxide semiconductor sensors each offer distinct strengths depending on the application, balancing sensitivity, real‐time capability, and ease of measurement.

Altogether, current evidence positions methane as a molecule of potential clinical relevance beyond what is understood today. Continued efforts to standardize measurement approaches, investigate potential mechanisms, and contextualize physiological relevance will be essential to assess its future role in both a gastrointestinal and possibly extra‐intestinal context.

## Author Contributions

M.K., M.A., and B.B. conceptualized the study and defined the scope of the topics. M.K., M.B., N.N., and R.P.‐L. contributed to writing the manuscript.

## Conflicts of Interest

M.K., M.B., I.M., N.N., R.P.‐L., M.A., and B.B. are employees of Owlstone Medical Ltd., a company specializing in breath‐based diagnostics and measurement technologies, including those relevant to this review.

## Data Availability

Data sharing not applicable to this article as no datasets were generated or analyzed during the current study.
